# Developing Cost-Effective Field Assessments of Carbon Stocks in Human-Modified Tropical Forests

**DOI:** 10.1371/journal.pone.0133139

**Published:** 2015-08-26

**Authors:** Erika Berenguer, Toby A. Gardner, Joice Ferreira, Luiz E. O. C. Aragão, Plínio B. Camargo, Carlos E. Cerri, Mariana Durigan, Raimundo C. Oliveira Junior, Ima C. G. Vieira, Jos Barlow

**Affiliations:** 1 Lancaster Environment Centre, Lancaster University, Lancaster, United Kingdom; 2 Stockholm Environment Institute, Stockholm, Sweden; 3 International Institute for Sustainability, Rio de Janeiro, Rio de Janeiro, Brazil; 4 Embrapa Amazônia Oriental, Belém, Pará, Brazil; 5 College of Life and Environmental Sciences, University of Exeter, Exeter, United Kingdom; 6 Tropical Ecosystems and Environmental Sciences Group (TREES), Remote Sensing Division, National Institute for Space Research—INPE, São José dos Campos, São Paulo, Brazil; 7 Centro de Energia Nuclear na Agricultura, Universidade de São Paulo, Piracicaba, São Paulo, Brazil; 8 Departamento de Ciência do Solo, Universidade de São Paulo, Escola Superior de Agricultura Luiz de Queiroz-Esalq, Piracicaba, São Paulo, Brazil; 9 Embrapa Amazônia Oriental, Núcleo do Médio Amazonas, Santarém, Pará, Brazil; 10 MCT/Museu Paraense Emílio Goeldi, Belém, Pará, Brazil; University of Oxford, UNITED KINGDOM

## Abstract

Across the tropics, there is a growing financial investment in activities that aim to reduce emissions from deforestation and forest degradation, such as REDD+. However, most tropical countries lack on-the-ground capacity to conduct reliable and replicable assessments of forest carbon stocks, undermining their ability to secure long-term carbon finance for forest conservation programs. Clear guidance on how to reduce the monetary and time costs of field assessments of forest carbon can help tropical countries to overcome this capacity gap. Here we provide such guidance for cost-effective one-off field assessments of forest carbon stocks. We sampled a total of eight components from four different carbon pools (i.e. aboveground, dead wood, litter and soil) in 224 study plots distributed across two regions of eastern Amazon. For each component we estimated survey costs, contribution to total forest carbon stocks and sensitivity to disturbance. Sampling costs varied thirty-one-fold between the most expensive component, soil, and the least, leaf litter. Large live stems (≥10 cm DBH), which represented only 15% of the overall sampling costs, was by far the most important component to be assessed, as it stores the largest amount of carbon and is highly sensitive to disturbance. If large stems are not taxonomically identified, costs can be reduced by a further 51%, while incurring an error in aboveground carbon estimates of only 5% in primary forests, but 31% in secondary forests. For rapid assessments, necessary to help prioritize locations for carbon- conservation activities, sampling of stems ≥20cm DBH without taxonomic identification can predict with confidence (R^2^ = 0.85) whether an area is relatively carbon-rich or carbon-poor—an approach that is 74% cheaper than sampling and identifying all the stems ≥10cm DBH. We use these results to evaluate the reliability of forest carbon stock estimates provided by the IPCC and FAO when applied to human-modified forests, and to highlight areas where cost savings in carbon stock assessments could be most easily made.

## Introduction

Climate mitigation activities that aim to avoid further deforestation and forest degradation, such as REDD+, could help reduce annual greenhouse gas emissions by 10–12% [1,2]. Payments to support these forest conservation activities have been claimed to be the most cost-effective way of mitigating climate change [3], and could potentially also deliver a suite of desirable environmental and social co-benefits, including biodiversity conservation, soil protection and water provision [4–6]. However, establishing lasting and credible carbon finance schemes requires countries to develop robust, transparent and verifiable systems for assessing and reporting changes in forest carbon [7]. Under the UN-led REDD+ process, countries are expected to follow the carbon accounting guidelines established by the Intergovernmental Panel on Climate Change (IPCC) [8], which suggest three levels, or tiers, of increasing methodological complexity to assess forest carbon stocks: Tier 1 uses global estimates of forest stocks, Tier 2 uses regional or country-level estimates of forest carbon, and Tier 3 uses data from local field and remote sensing carbon assessments. However, as of 2009, only 3% of tropical countries had developed adequate capacity to assess forest carbon stocks [9] and, since then, little progress has been observed [10]. This capacity gap is especially noticeable when considering field assessments (i.e. Tier 3), which are essential to understand changes in carbon stocks following human disturbance (e.g. selective logging, understory fires, and edge effects [11,12]), and to calibrate remote sensing estimates of forest stocks [13]).

Any effort to address this capacity gap requires the development of cost-effective guidelines to provide a basis of reliable and replicable assessments of forest carbon stocks. The lack of such guidelines undermines the ability of countries to secure long-term carbon finance for forest conservation programs [14]. Furthermore, cost-effective carbon stock assessments can reduce transaction costs of mitigation programs, making them more attractive to new investors, which are urgently needed to scale-up carbon-conservation efforts. Natural scientists can play a crucial role in helping the development of cost-effective carbon assessments under Tier 3. However cost-effectiveness studies are generally rare in the natural sciences (but see [15–19]), especially in the tropics, and none to date have focused on quantifying the costs of estimating forest carbon stocks through field assessments.

The development of cost-effective guidelines for forest carbon accounting is particularly important in human-modified tropical forests, which are undergoing some of the highest rates of deforestation and forest degradation in the world [20,21]. These forests include logged, burned and fragmented primary forests as well as secondary forests regenerating on cleared land, and are an increasingly prevalent feature across tropical forest landscapes [22–24]. Activities to avoid further degradation and losses of carbon from human-modified forests represent an important opportunity for emission reductions and forest conservation, especially in regions that lack large areas of undisturbed forests [25]. Despite their often high carbon-conservation value [26,27] and their risk of conversion to agricultural land-uses [20], human-modified tropical forests are rarely the focus of conservation initiatives and of research activities.

Here we provide the first assessment of the costs, in terms of both money and time, of conducting a comprehensive field assessment of carbon stocks in human-modified tropical forests. We sampled eight components from four different carbon pools (aboveground, dead wood, litter and soil) in 224 forest plots distributed across two regions of the Brazilian Amazon. We present the overall costs (time and money invested) of sampling individual components of the total carbon stocks. Although these costs are specific to our study area and sampling design, the relative costs of sampling different components of the total carbon stocks should be applicable to other tropical regions. We then undertake a cost-effectiveness evaluation of field assessments of forest carbon stocks focusing on three specific objectives. First, we examine how much each individual component (i.e. trees of different size classes, coarse and fine dead wood, litter, and soil) contributes to total estimated carbon stocks and how variable is that contribution across replicate samples. Second, we use this information to identify cost-effective sampling strategies by asking how our ability to estimate forest carbon stocks in different types of human-modified forests is affected by a) only sampling large live stems, b) only sampling a subset of large live stems, c) not identifying large live stems, and d) using freely available estimates of forest carbon stocks from the Food and Agriculture Organization of the United Nations (FAO) and from the IPCC (a Tier-2 approach). Finally, we evaluate the most cost-effective way for landowners and conservation practitioners to predict if a targeted area is carbon-rich or carbon-poor. We discuss our results by addressing practical aspects relevant to the establishment of carbon-conservation projects in human-modified tropical forests, providing guidance on which tier should be employed when sampling each individual carbon pool.

## Methodology

### Ethics statement

Research permits for plots situated inside the Floresta Nacional do Tapajós were provided by Instituto Chico Mendes de Conservação da Biodiversidade (24164–2). For plots located in rural properties, we received authorization for fieldwork development from each individual landowner. Occasionally, field sampling involved measurement and identification of naturally occurring protected or endangered plant species (e.g. *Bertholletia excelsa* and *Euxylophora paraensis*), however none of these were damaged or killed, following the standard measurement procedures of all other plant species.

### Study areas

**Fig 1:**
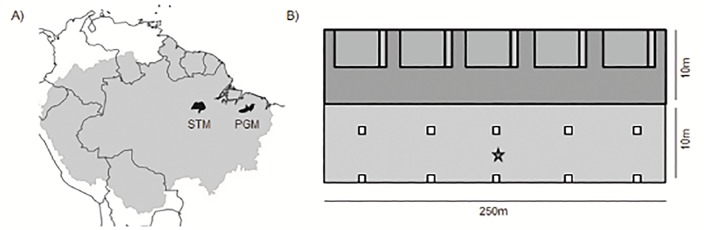
Sampling design. A) Location of the two study regions, Paragominas (PGM) and Santarém-Belterra (STM), within the Amazon Basin (in light gray), B) Carbon stocks assessment: Large dark gray rectangle—survey of live and dead trees and palms ≥10cm DBH and live lianas ≥10cm diameter at 1.3m from the main rooting point. Gray rectangles and small light gray rectangles attached– 5x20m subplots for identification and measurement of all live and dead trees and palms ≥2–9.9cm DBH and live lianas ≥2–9.9cm diameter at 1.3m from the main rooting point. Measurement of coarse woody debris (≥10cm diameter in at least one extremity) was also carried out in the 5x20m subplots. Small light gray rectangles– 2x5m subplots for fine woody debris sampling (≥2–9.9cm diameter in at least one extremity). Squares– 0.5x0.5m quadrats for leaf litter sample. Underneath the first row of litter sampling (5m away from the plot), composite soil samples were collected at three different depths: 0–10, 10–20 and 20-30cm. Star– 30x30cm trench for sampling of soil bulk density to calibrate soil carbon stocks.

Carbon stock assessments were carried out in two regions of eastern Amazon located c. 800km apart (Fig 1A): Santarém-Belterra (2° 26’S, 54° 42’W) and Paragominas (2° 59’S, 47° 21’W). During the past 40 years these municipalities have experienced high rates of land-use change and now present a mosaic of agricultural and forested lands [28]. In each municipality, 18 study catchments (c. 5000 ha each) were selected along a gradient of remaining forest cover (6–100% in Paragominas and 10–100% in Santarém). In every catchment, study plots (10x250m; 0.25ha) were randomly located in evergreen non-flooded forests, applying a minimum separation between them of 1500m to maximize spatial independence. The number of plots per catchment varied according to the amount of remaining forest cover of each catchment: more plots were established in highly forested catchments than in catchments with little forest left; following a density rule of 1 plot per 400 ha. As a result of the random distribution, our plots comprised a range of undisturbed and varyingly disturbed primary forests (e.g. logged, burned) as well as secondary forests (ranging from 6 to over 22 years old). We sampled a total of 224 plots (117 in Paragominas and 107 in Santarém), distributed across an area of more than three million hectares. Using a combination of ground assessment of past human-disturbance (e.g. logging debris, charred stems, and charcoal on the forest floor) and a visual analysis of a 20 years chronosequence of Landsat images, plots were classified into one of three categories: undisturbed primary forests, disturbed primary forests, or secondary forests (Table 1; see [26] for more details on plot classification).

**Table 1:** Number of sampled plots (0.25ha) in each study region.

Forest Class	Paragominas	Santarém
Undisturbed primary forest	13	17
Disturbed primary forest	88	57
Secondary forest	16	33
**Total**	**117**	**107**

### Field sampling and biomass estimates

In every plot we assessed four different carbon pools from the five determined by the IPCC guidelines [8]: 1) Aboveground (live trees, palms and lianas); 2) Dead Wood (dead trees and palms, as well as coarse woody debris); 3) Litter (fine woody debris and leaf litter); and 4) Soil (0–30 cm depth). The belowground carbon pool, composed by coarse roots, was not assessed. Considering both regions, we measured a total of 30,100 live and dead stems of trees, palms and lianas ≥10cm DBH (DBH = 1.3m from the ground); 39,893 live and dead stems of trees, palms and lianas 2–9.9cm DBH; 8,601 pieces of coarse woody debris; undertook 1,120 samples of fine woody debris; 2,240 litter samples and 4,704 soil samples (Fig 1B). We assumed carbon to account for 50% of biomass content of all components [8].

#### Vegetation

All trees and palms ≥10cm DBH were measured and identified to species level, as well as all lianas ≥10cm diameter at 1.3m from the main rooting point (Fig 1B). Trees and palms 2–9.9cm DBH and lianas 2–9.9cm diameter were sampled along 5 subplots (5x20m). Dead trees and palms, irrespectively of the size, also had their height estimated. All stems of individuals with bifurcations <1.3m height were measured. To estimate the biomass of live trees and palms we used Chave’s equation for tropical moist forests, incorporating both DBH and species specific wood density [29]. Data on species wood density was collated from The Global Wood Density Database [30], filtering values only from tropical regions of South America. When wood density data on a given species was not available, genus average was used and when that was not present, we used a family average. To calculate the biomass of lianas we applied an allometric equation developed in eastern Amazon [31]. Biomass of dead trees was estimated using Hughes’ equation [32], while Cummings’ equation was applied to assess the biomass of dead palms [33].

#### Coarse Woody Debris

Coarse woody debris (CWD), pieces of dead wood ≥10cm diameter on at least one end, were sampled in 5 subplots (5x20m) distributed along the plots (Fig 1B). We measured total length and diameter at both ends of all pieces of CWD, as well as all bifurcations ≥10cm diameter at one extremity. In the case of CWD resembling planks (generally resulting from logging processing), thickness was also determined by taking two measures of each side. All pieces had their level of decomposition assessed and were classified into five different classes, ranging from recently dead to completely soft, rotten, crumbling wood; following Harmon & Sexton [34]. As CWD can often present severe damage due to the fall, we categorized all pieces into five classes: no damage, <25%, 25–50%, 50–75%, >75% damage. Planks had their volume estimated through the formula of the cube, while for all other CWD we used the Smalian’s formula [35]. We discounted the percent damage from the final volume of each piece. Biomass of CWD was estimated by multiplying the final volume of each piece by the density of its decomposition class, following Keller’s approach in the same region [36].

#### Fine Woody Debris

We sampled all fine woody debris (FWD), necromass between 2–9.9cm diameter on at least one end, present in a smaller area of the subplots (2x5m; Fig 1B). All pieces of fine woody debris were grouped together and their combined fresh weight was measured in the field, after which a sub-sample (<1kg) was separated and weighed before being transported to a local laboratory, where it was oven-dried to constant weight. The biomass of FWD was calculated based on the wet-to-dry weight ratio of the sub-samples.

#### Leaf Litter

Five paired samples of leaf litter were collected per plot using a 0.5x0.5m quadrat. Twigs ≥2cm diameter at one extremity were excluded from the samples, as they constitute fine woody debris. Each paired sample was taken 50m apart from each other, while samples in a pair were separated by 5m (Fig 1B). Litter samples were taken to a local laboratory and oven-dried to constant weight.

#### Soil

A trench (0.3x0.3m) was opened in the center of all plots to assess soil bulk density. We used volumetric rings to collect two undisturbed soil cores at three depths (0-10cm, 10-20cm, 20-30cm) and calculated soil bulk density by dividing the soil mass of each soil core by the volume of the ring. Composite soil samples were taken at the same three depths at five sampling points separated by 50m intervals along each plot (Fig 1B). Soil samples were air-dried and sieved (2mm mesh) in a laboratory. Sub-samples of soil (10g) were then removed and further sieved (0.149 mesh). An elemental analyzer was used to determine carbon percentage by dry combustion. Soil carbon stocks were estimated by multiplying the carbon content of each layer by layer thickness (10cm) and soil bulk density. To obtain the total soil carbon stocks of each plot we first averaged the stocks of each layer across the five sampling points and then we summed the average stocks of all the three layers.

### Sampling time and costs

Monetary costs of sampling each component of the total forest carbon stocks (i.e. vegetation, CWD, FWD, litter and soil) were calculated in U$ dollars/hectare and comprised field and laboratory expenses, including both equipment (consumables and chemicals) and labor. As most equipment has a life-expectancy of more than a single 1-ha plot assessment, we divided equipment purchase costs by their expected lifespan (e.g. a DBH tape costs U$45, but lasts for 15 1-ha plot assessments; yielding U$3 dollars/hectare). Costs of heavy-duty laboratory equipment, such as ovens to dry samples to constant weight and elemental analyzers to assess soil stocks, were not incorporated in any analyses, as we assumed that governmental, academic or corporate institutions conducting carbon inventories are more likely to establish partnerships with local laboratories than to fully build and equip a new one. Although accommodation and transport costs are a significant part of any field assessment of carbon stocks in tropical rainforests, these were also excluded from analyses, as they will vary considerably according to each particular location and logistical arrangements (e.g. transport costs can involve car, boat and/or helicopter rental in addition to fuel). Labor costs were divided into four levels, reflecting worker skills and salaries paid in our study regions: (i) field assistants, U$16.5/day, (ii) laboratory technician (only necessary for soil sampling due to the equipment used), U$35.75/day, (iii) field leader (e.g. graduate students responsible for recording and organizing sampled data), U$55/day and (iv) parabotanists (experts able to identify Amazonian plants to species level both in the field and at a herbarium using Latin nomenclature), U$121/day. Whilst the absolute values will differ, the ratio of these salary levels is likely to be comparable for other tropical forest regions. Time effort was calculated as the number of minutes spent in the field and in the laboratory when assessing each part of the stocks in our study. To calculate the costs of measuring only a subset of the live stems, we excluded equipment costs, which represented only 7% of the overall vegetation sampling costs. Therefore, for comparative analyses between different subsets of live stems we considered costs to consist only of personnel time, calculated as the average time to measure and identify a single stem multiplied by the total number of stems in each subset plus a fixed amount of time (120min) necessary to walk a 1-ha plot verifying if there are any individuals to be measured within the plot’s limits. Although our own study plots were only 0.25ha, all costs were calculated for 1-ha plot assessments, as this is the most common plot size used for carbon assessments in the tropics (e.g. [37,38]). Finally, costs related to digitizing and analyzing data as well as of writing concluding reports were also excluded from all analyses: we focused solely on the costs of field and laboratory activities.

### Data analysis

The contribution of each individual component to total forest carbon stocks was defined as the average carbon content of any given component over the total sampled stocks in each study plot. We used the coefficient of variation (ratio of standard deviation to mean) to assess the variability in carbon stored in each component between forest plots. Although live and dead plants were sampled together, they were separated in analyses of relative stock contributions, as they belong to functionally different carbon pools: live vegetation constitutes the aboveground carbon pool; while dead trees and palms are part of the dead wood pool, representing committed emissions from the decomposition of organic material [8]. To check if results of the relative contribution of each individual component and their coefficient of variation were similar between the two study regions, we performed a Spearman’s rank correlation test. To evaluate if the carbon stored in large live stems (i.e. ≥10 cm DBH) is a good predictor of the carbon stored in all other components of the total stock, we used simple linear regressions.

To compare the relative loss of accuracy (i.e. error) associated with using simplified sampling protocols, we divided our full dataset (i.e. all live stems ≥10cm DBH identified to species level) into subsets according to plant size: stems ≥20cm DBH, stems ≥30cm DBH, stems ≥40cm DBH, and stems ≥50cm DBH. We also estimated the stocks of all the above subsets using a single value for wood density specific to eastern Amazonian forests (0.639; [39]), simulating field protocols where stems are not identified (not unusual due to a lack of trained field botanists in most tropical forest areas). As many assessments of forest carbon stocks are based on default national or regional values of aboveground carbon stocks (Tier 2), we also compared our results to the FAO estimate of aboveground carbon for Brazilian forests [40], and the IPCC estimate of aboveground stocks for North and South American tropical rainforests [8]. Error in the estimation of stocks was calculated as the average difference between our best measure (all stems ≥10cm DBH identified to species level) and each of the other individual measures. We present the absolute values of the errors, regardless of their direction (i.e. over or underestimation). Finally, as landowners and conservation practitioners may want to rapidly assess if an area is worth the investment of a carbon offsetting program (i.e. carbon-rich, and therefore priority for conservation measures), we used linear regression to assess, across all plots, the ability of each subset of live stems to predict the carbon stocks present in all large live stems combined.

Analyses were separated into three hypothetical scenarios of carbon stocks assessments in human-modified tropical forests: (i) no *a priori* information about the disturbance and clearance history of the forest (i.e. all our sampled plots pooled together), (ii) primary forests only (regardless of type or level of disturbance), and (iii) secondary forests only. The first represents the reality of most carbon assessments in human-modified landscapes, where there is no previous or reliable knowledge to confidently separate areas of highly disturbed primary forests from areas of mature secondary forests (a distinction that can be almost impossible without high-resolution time-series satellite data, or detailed historical land management records). The second and third scenarios exemplify assessments in areas where the forest class is known (e.g. carbon inventories executed inside logging concessions or inside restoration areas). All analyses were carried out in R version 2.15.1 [41]. We deposited all the data used in this paper in Figshare: http://dx.doi.org/10.6084/m9.figshare.1319500.

## Results

### Financial costs and time effort

If we had assessed each component of the total forest stocks separately, the overall cost of estimating carbon stocks in 224 0.25ha forest plots would have been c. U$364,000. By combining the sampling of some components (i.e. sampling large and small stems together, CWD together with FWD, and soil together with litter) we reduced the monetary costs by 18%, yielding a total investment of U$298,000. However, this amount does not include costs related to transport, accommodation, subsistence, health insurance and general field emergencies (which, when all combined, yielded an additional sum of approximately U$168,000). Since carbon assessments in the field are generally performed in 1-ha plots, all the following results have been standardized to dollars per hectare.

**Fig 2:**
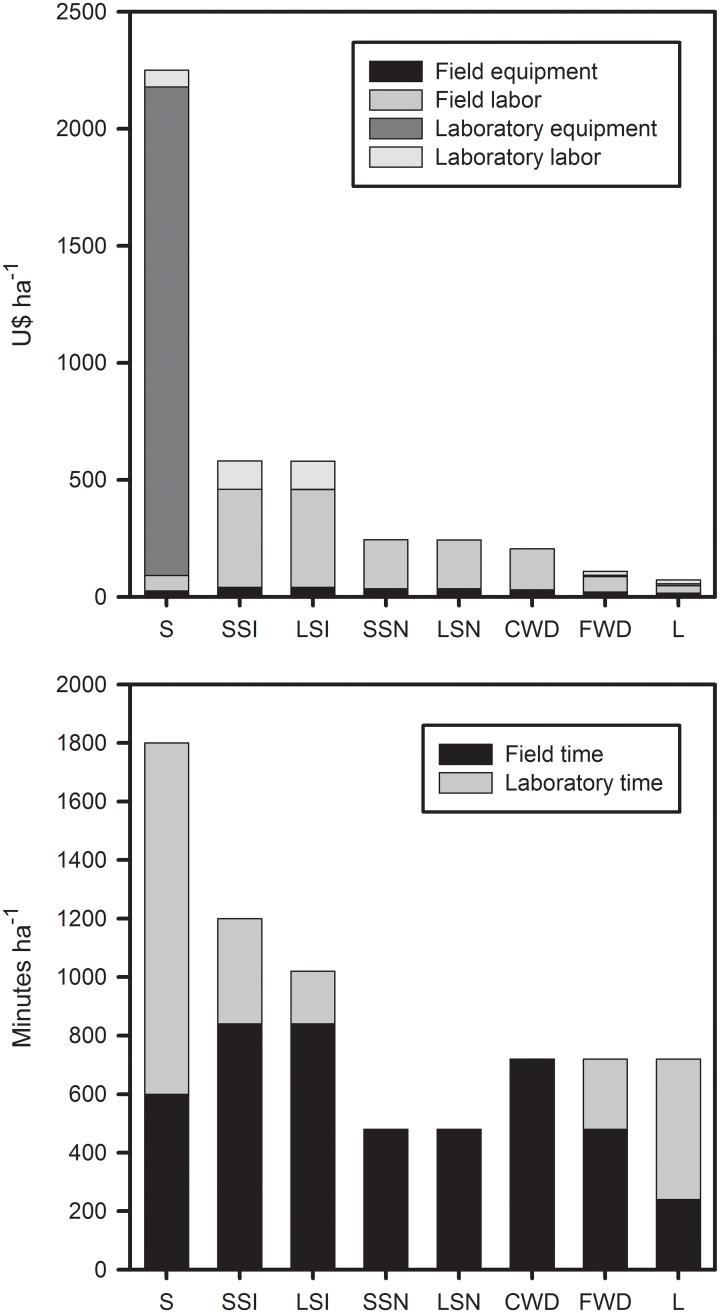
Financial costs and time spent sampling different components of the total carbon stocks. S = Soil 0-30cm, SSI = Small stems (2–9.9cm DBH) identified to species level, LSI = Large stems (≥10cm DBH) identified to species level, SSN = Small stems without species identification, LSN = Large stems without species identification, CWD = Coarse woody debris, FWD = Fine woody debris, L = Leaf litter. As live and dead stems were sampled together, it is impossible to disentangle their specific costs in this analysis.

Soil was by far the most costly component of the forest carbon stocks to be assessed, both in terms of financial costs and time effort (Fig 2). Most of this high monetary cost is related to laboratory material and equipment (consumables and chemicals) needed to analyze soil carbon, even though the capital costs of purchasing heavy-duty equipment, such as an elemental analyzer, were excluded. Carbon assessments of vegetation (including both large and small stems) were 58% cheaper without species identification than when all stems were identified (Fig 2), due to the high costs associated with hiring experienced taxonomic experts. Although coarse woody debris, fine woody debris and litter required similar amount of time investment according to our sampling design (Fig 2), the estimation of the carbon content of coarse woody debris was 47% and 65% more expensive than that of fine woody debris and litter, respectively.

### Contribution and variability of different components

**Fig 3:**
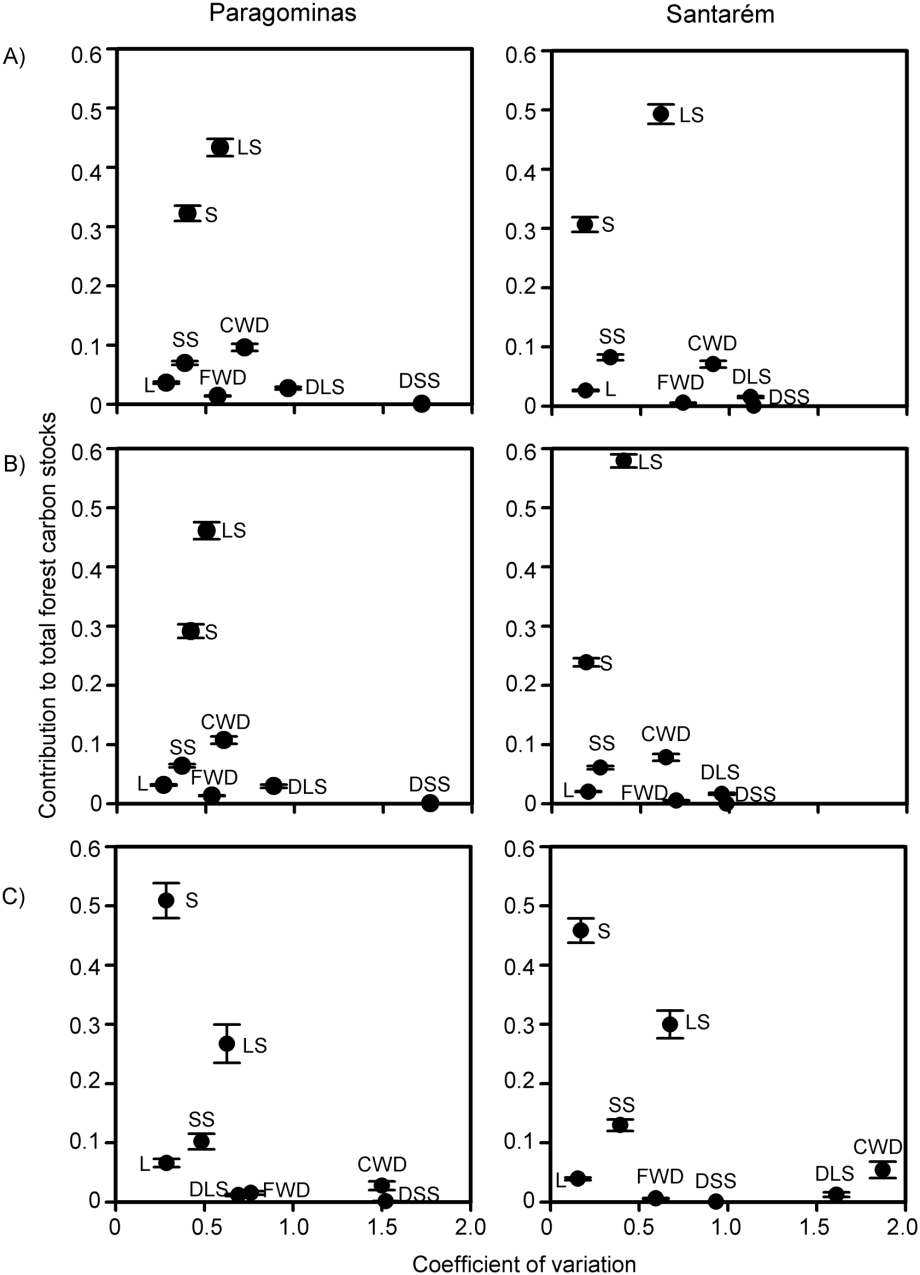
Average contribution and variation of different components of the total forest carbon stocks. Results are separated into three hypothetical scenarios of carbon stock assessments in human-modified tropical forests: A) No *a priori* information of forest class; B) Primary forests only—includes undisturbed and disturbed primary forests; and C) Secondary forests only. LS = Large live stems (≥10cm DBH), SS = Small live stems (2–9.9cm DBH), S = Soil 0-30cm, CWD = Coarse woody debris, FWD = Fine woody debris, L = Leaf litter, DLS = Large dead stems, DSS = Small dead stems.

Large live stems (≥10cm DBH) stored the greatest amount of carbon in areas without any prior information regarding the type of forest—i.e. all forest classes pooled together—(43–49%; Fig 3A) and in areas containing only primary forests (46–58%; Fig 3B). However, in secondary forests (Fig 3C) soil in the 0-30cm profile had a higher contribution to total stocks (46–51%) than large live stems (27–30%). Regardless of the hypothetical scenario of carbon assessment in human-modified tropical forests, the combined contribution of all other components (i.e. CWD, FWD, litter, small live stems, small dead stems, and large dead stems) represented less than 25% of the total stocks. The carbon stock of large live stems was highly variable between sample plots in any given scenario (coefficient of variation ≥0.40), although the components of the dead wood carbon pool (CWD and small and large dead stems) consistently presented a higher coefficient of variation than large live stems. Both the relative contribution and the variability of individual stock components were highly correlated between study regions when considering all scenarios separately (relative contribution: rho >0.95; variability: rho >0.80; Table A in S1 File).

### Using large live stems to predict the carbon stored in all other components

In both regions and across all hypothetical scenarios of carbon assessments in human-modified tropical forests, the amount of carbon stored in large live stems (≥10cm DBH) was a poor predictor of the carbon stocks of all other components (Figs A–G in S1 File).

### Errors and costs of measuring only subsets of large stems

**Fig 4:**
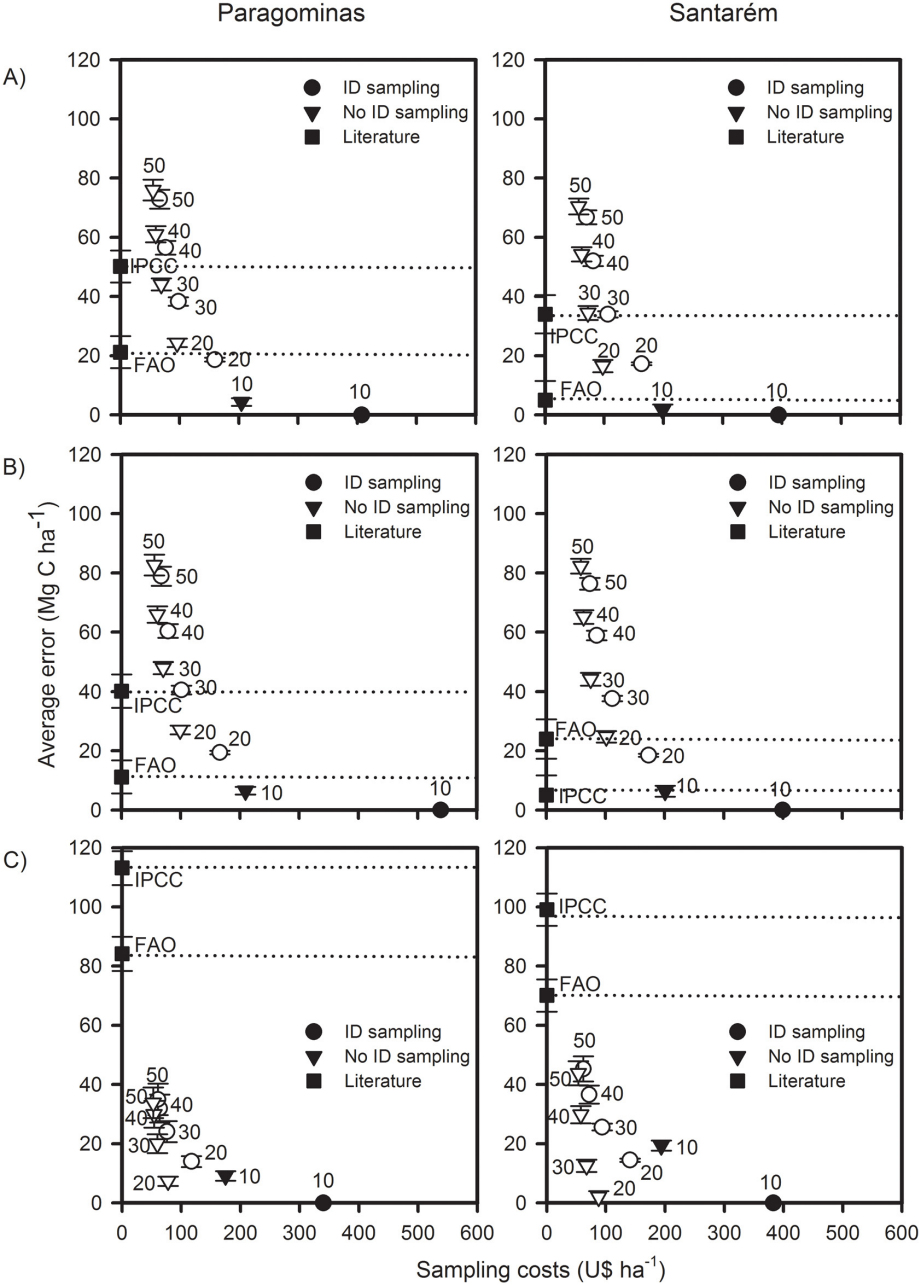
The costs and errors of simplifying carbon sampling protocols. Relationship between the average error of different estimates of carbon stored in large live stems and the costs of sampling a 1-ha plot. Results are separated into three hypothetical scenarios of carbon stock assessments in human-modified tropical forests: A) No *a priori* information of forest class; B) Primary forests only—includes undisturbed and disturbed primary forests; and C) Secondary forests only. Filled symbols indicate estimates of carbon stocks present in all stems ≥10cm DBH, whereas open symbols represent carbon estimates of subsets of large live stems. The dotted lines indicate the average error of both the IPCC and FAO estimates.

When estimating carbon stocks of large live stems only (Fig 4), there was a reduction in costs but an increase in error associated with the increase of the DBH cut-off point of each sampled subset (e.g. the error associated with sampling only stems ≥40cm DBH is larger than the error associated with sampling only stems ≥30cm DBH). Across all scenarios of carbon assessments in human-modified tropical forests, there is an average reduction in U$257 per hectare when protocols sample only stems ≥20cm DBH instead of all stems ≥10cm DBH, accompanied by an average error increase of 17 Mg C ha^-1^; whereas the reduction in costs for simplifying even further the sampling protocol and measuring and identifying only stems ≥50cm DBH incurs in an economy of just an extra U$87 per hectare with an average error increase of 46 Mg C ha^-1^.

### Errors and costs of not identifying large stems

By measuring but not identifying stems ≥10cm DBH, costs of carbon assessments can be reduced by 51%, while the average error associated with this simplified protocol is 2.96 Mg C ha^-1^, 6.42 Mg C ha^-1^ and 14.22 Mg C ha^-1^ respectively for areas without *a priori* information of forest class, in areas of primary forests only, and in areas of secondary forests only. These errors represent 3%, 5%, and 31% of the stocks contained in large stems in the same scenarios (Fig 4). In both regions, sampling of all large live stems (≥10cm DBH) without taxonomic identification presented a smaller error than that incurred when measuring and identifying only stems ≥20cm DBH (in average 9.18 Mg C ha^-1^ lower), leading to an extra average cost of only U$44 per sampled hectare.

### Errors of using freely available estimates of forest carbon stocks

In general, the FAO default estimate of carbon stocks stored in large live stems performed better than the IPCC one (Fig 4). For example, in areas without *a priori* information of forest class, the FAO estimate had an average error of only 21.16 Mg C ha^-1^ in Paragominas and 5.02 Mg C ha^-1^ in Santarém; while the IPCC estimate had an average error of 50.16 Mg C ha^-1^ and 34.02 Mg C ha^-1^, respectively. Across the two study regions, both estimates presented lower associated errors in primary forests than in secondary forests. Overall, better estimates of forest stocks can be obtained by either measuring and identifying all stems ≥20cm DBH or by only measuring stems ≥10cm DBH, rather than by using either the FAO or the IPCC default values (Fig 4).

### Predicting if a forest is carbon-rich

**Fig 5:**
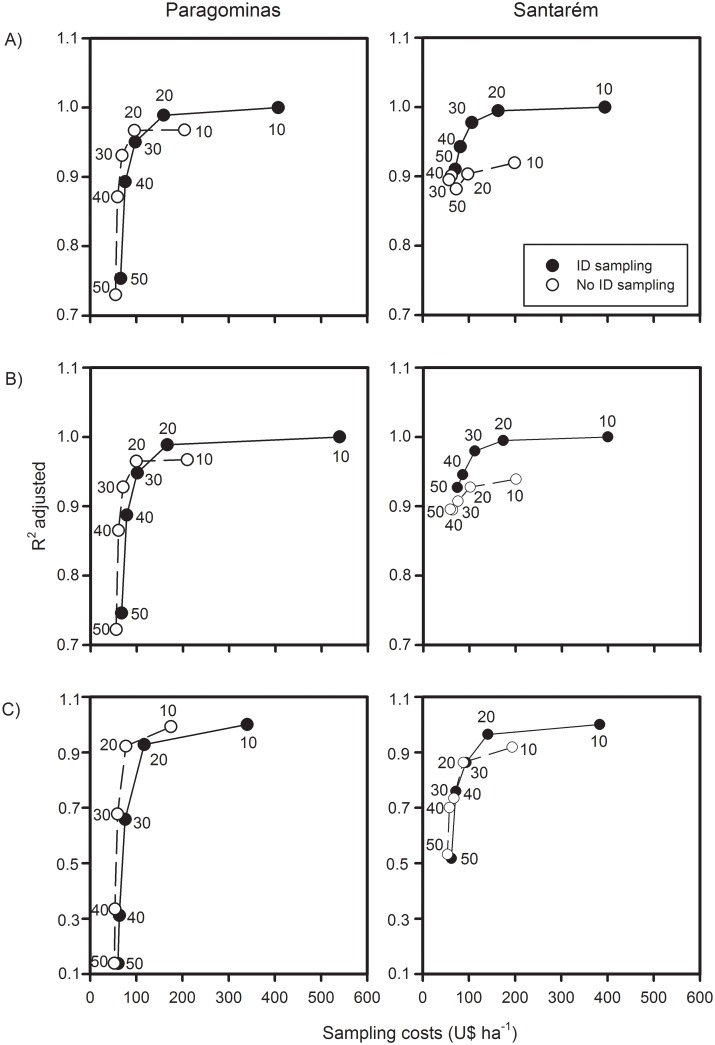
Predicting if a forest is carbon-rich or carbon-poor. Relationship between the coefficient of determination (R^2^) of different subsets of large live stems and the costs of sampling a 1-ha plot. Results are separated into three hypothetical scenarios of carbon stock assessments in human-modified tropical forests: A) No *a priori* information of forest class; B) Primary forests only—includes undisturbed and disturbed primary forests; and C) Secondary forests only. The coefficient of determination was obtained through linear models applied to calculate the power of each subset of stems to predict the carbon stocks present in our best measure of large stems stocks (all stems ≥10cm DBH identified to species level).

Across both regions and irrespective of the forest class assessed, measurement of stems ≥30cm DBH without species identification could predict with confidence (R^2^ ≥0.7) if a forest is carbon-rich (Fig 5). By only surveying stems ≥30cm DBH, rapid carbon assessments of areas to be set aside for conservation could reduce costs by 80% when compared to measuring and identifying all stems ≥10cm DBH. More conservative rapid assessments could focus on measuring stems ≥20cm DBH to predict if a forest is carbon-rich (R^2^ >0.85 under all three hypothetical scenarios of carbon inventories in human-modified tropical forests), and still have costs 74% lower than surveying and identifying to species level all stems ≥10cm DBH.

## Discussion

Our results indicate that significant cost savings can be achieved in field assessments of forest carbon stocks with minimal loss of accuracy. In particular, the cost of carbon stock assessments in human-modified tropical forests can be greatly reduced if only large live stems (≥10cm DBH) are sampled (Fig 2). This component stores great amounts of carbon and is also highly variable between human-modified forest sites (Fig 3), indicating a high sensitivity to anthropogenic disturbance. However, sampling of large live stems tells us little about all the other components of the forest carbon stocks (R^2^_adjusted_ <0.52 for all regressions; Figs A–G in S1 File). To further reduce costs, sampling protocols can opt to exclude stem identification and use instead regional estimates of wood density in allometric equations used to estimate stocks, with only minimal loss of accuracy (Fig 4), particularly in primary forests. Finally, rapid carbon assessments aiming only to identify carbon-rich forests (rather than to estimate actual stock levels), can save 74% of monetary costs by focusing only on measuring stems ≥20cm DBH without taxonomic identification (Fig 5). We discuss these results by addressing four questions relevant to the establishment of carbon-conservation projects in human-modified tropical forests: (i) Does *a priori* information about disturbance and clearance history of an area affect guidance on cost-effective carbon sampling protocols? (ii) How reliable are the IPCC and the FAO default carbon values in estimating stocks of human-modified tropical forests?; (iii) Which components of the total forest carbon stocks should be measured to maximize the cost-effectiveness of field assessments?; and (iv) How can sampling of large live stems be more cost-effective?

### How important is *a priori* information about disturbance and clearance history?

Overall, our results about the importance and the variability of each stock component, as well as results on ways of simplifying sampling protocols, were consistent both between and within regions, regardless if relative to areas with or without any *a priori* information of forest disturbance and clearance. This indicates that our general recommendations hold irrespective of whether information is available on the mix of forest types in a given landscape of interest. This is particularly encouraging given that the discrimination between highly disturbed primary forests and old secondary forests can be difficult, or even impossible, in situations where there is a lack of detailed satellite data or when forest disturbance or clearance happened prior to imagery baseline. Even when comprehensive satellite imagery is available, both automated and visual analyses of images can be time and resource consuming, requiring trained personnel. Such staff requirements may be difficult to meet in many tropical forest countries [9,10].

It is important to note, however, that field surveys of signs of past human disturbance, such as logging debris or charred stems (Figs H–I in S1 File), can be extremely valuable in providing a better understanding of what external stressors could affect a targeted conservation area, helping conservation practitioners to delineate measures to both minimize the threats posed by future human impacts and to rehabilitate already degraded areas. Field surveys of past human disturbance need not incur extra costs, as they can be conducted together with carbon inventories by simply observing evidence of fire or logging in a systematic way while measuring stocks (Figs H–I in S1 File) [26,42].

### How reliable are the IPCC and FAO carbon estimates in human-modified tropical forests?

If freely available regional and national estimates of aboveground carbon stocks, such as those from the IPCC [8] and FAO [40], were able to provide accurate estimates of carbon stocks in human-modified tropical forests, carbon-conservation programs could be made much cheaper by not conducting field assessments at all. However, our results show that both the IPCC and FAO estimates of carbon stocks performed poorly, leading to errors as high as 113 Mg C ha^-1^ in areas of secondary forest. Such discrepancies are not surprising, given that these estimates were developed based on data from undisturbed primary forests. Our results therefore clearly demonstrate the importance of carrying out field assessments of carbon stocks in human-modified tropical forests, rather than relying on existing look-up tables. This is particularly important when considering that much of the carbon inventories performed to date have focused on undisturbed forests only (e.g. [38,43,44]), despite the fact that the area occupied by disturbed primary and secondary forests already account for over 500 million hectares of the remaining forest cover in the tropics [45].

### Which carbon stock components should be measured to maximize the cost-effectiveness of field assessments?

For carbon assessments in human-modified tropical forests located on non-peaty soils, our results indicate that sampling should focus only on large live stems (≥10cm DBH). From all the sampled components of the total forest carbon stocks, large stems stored the greatest amount of carbon and were particularly sensitive to human-induced disturbance (Table 2). By focusing sampling protocols only on large live stems, field assessments in 1-ha plots could be made 85% cheaper and 84% quicker, thus substantially increasing the possibility of carrying out field assessments over larger areas. Additionally, the fact that large live stems form the most important component of the total forest carbon stocks also means that new and promising tools for carbon estimation that focus on large stem biomass to calculate forest stocks, such as airborne LiDAR, can be valuable when assessing carbon stocks over very large areas (e.g. municipality-wide). However, although the use of airborne LiDAR is cheaper than establishing and sampling plots distributed over millions of hectares [46], it is unlikely to be suitable for small carbon-conservation projects, due to the high monetary costs associated with sampling a small area [47].

**Table 2:** Recommended sampling approaches to assess carbon stocks in human-modified tropical forests. Recommendations are based on the results of this study, following the IPCC three- tiers system for forest carbon assessments.

Carbon pool	Components	Recommendation
Aboveground	All living vegetation	**Tier 3** (large live stems) **and 1 or 2** (other components). The aboveground carbon pool stores the largest amount of carbon (excluding deep soils), is extremely sensitive to human disturbance and relatively easy to sample. Most of the carbon stored in this pool is in large stems (≥10cm DBH) and therefore we recommend these to be assessed on the field (Tier 3), while other components of the aboveground pool (e.g. stems <10cm DBH) should be assessed using estimates provided in the literature (Tier 1 or 2).
Dead Wood	Dead vegetation and coarse woody debris	**Tier 1 or 2.** The dead wood carbon pool contributes to less than 10% of the total carbon stocks. In addition, it has no value in terms of carbon sequestration or even long-term carbon storage, as it comprises decomposing material and therefore represents committed emissions.
Litter	Leaf litter and fine woody debris	**Tier 1 or 2.** Besides making little contribution to total forest carbon stocks, the litter carbon pool poses logistical difficulties as it requires use of laboratory facilities to oven dry and weight its components.
Soil	Soil carbon stocks and fine roots (<2mm)	**Tier 3** (peatlands), **1 or 2** (non-peaty soils). Despite making a large contribution to forest carbon stocks, soil carbon is extremely expensive and time-consuming to sample. This pool also requires the use of well-equipped facilities to conduct laboratory analysis, which are often difficult to find in tropical forested regions. Furthermore, in non-peaty soils, soil carbon does not appear to present much variability, indicating that it is relatively insensitive to human-induced disturbance.
Belowground	All living coarse roots	**Tier 1 or 2.** As the belowground carbon pool involves destructive sampling, thus incurring severe damage or even death to the trees, it cannot be included as part of a recommended field protocol. Besides, projects aiming to conserve forest carbon stocks will necessarily preserve live stems and, as a consequence, the live belowground pool will be preserved as well. Therefore, root/shoot equations available in the literature (e.g.[60–62]) can be used to estimate the amount of carbon stored in coarse roots.

Despite soil carbon being the most expensive and time-consuming component to sample, and one that varied very little across our 224 sampled plots, caution is needed in assuming that soil should not be measured as part of carbon stock assessments elsewhere (Table 2). First, we did not assess the deeper carbon pool, although we might expect the deeper soil to be more resistant to change than the first 30cm [48]. Second, responses may be different in places with distinct disturbance regimes: for example, logging intensities in Southeast Asian dipterocarp forests are much more severe than those in the Amazon [49], and we currently have little information on how soil carbon may change following logging in these forests. In particular, the assessment of soil carbon is strongly recommended in peatland forests, where it is known to be extremely sensitive to human disturbance [50,51] and to store up to three times more carbon than the aboveground pool [52]. Therefore, project developers and funders should be aware that carbon assessments in peatlands and intensively logged forests could require a higher initial investment to ensure an accurate, reliable and robust assessment of their carbon stocks. Finally, our results provide very little support for the sampling of small stems (<10cm DBH), coarse woody debris, fine woody debris and litter when establishing carbon-conservation projects—the combined contribution of all these components to total forest stocks was less than 25% (Table 2).

### How can sampling of large live stems be more cost-effective?

The salaries of experienced parabotanists represent 63% of the cost of sampling carbon stocks in vegetation and, as a consequence, any carbon assessment carried out without species-level identification will have much lower financial costs. Hence, on the strict perspective of carbon conservation, species identification would not be recommended. Nevertheless, stem identification will be important if a carbon-conservation project is also looking to maximize biodiversity co-benefits [5]. In addition species identification is advisable if one-off carbon assessments are to turn into longer-term monitoring of stocks: monitoring systems that chose to ignore species identification might fail to notice compositional changes, which in turn may lead to an erosion of the ecological integrity and resilience of the forest, often resulting in associated losses of carbon stocks [53]. For instance, following selective logging and understory fire events, primary forests are known to experience a significant increase in the abundance of pioneer trees, which store substantially less carbon than old-growth species [54–57]. An alternative to the identification of all large stems is to incorporate in sampling protocols the identification of a few distinctive pioneer tree species that are familiar to field technicians, but for which identification would not incur in extra time being spent on the field (e.g. *Cecropia spp* in the neotropics). Assessment of pioneers has the added advantage of indicating the presence of new disturbances, as pioneer species are characteristic of changing systems [58,59].

## Conclusion

The use of cost-effective guidelines in forest carbon assessments can potentially increase the appeal of carbon-conservation programs to new investors, who are much needed. The use of protocols that focus on assessing only the most relevant carbon pools (i.e. the ones that contribute the most to total stocks and are also highly vulnerable to environmental changes and human-induced disturbance) can greatly reduce both the complexity and costs of estimating forest carbon stocks, while sacrificing relatively little accuracy. Furthermore, the development of cost-effective guidelines for carbon assessments in human-modified tropical forests could considerably increase their chances of conservation. This is important as although human-modified forests do not have the same conservation value as undisturbed primary forests, they are the dominant feature of many regions of the humid tropics and, in many places, constitute the last remaining forests. Forest conservation continues to provide a huge opportunity for both climate mitigation and the conservation of biodiversity and ecosystem services. The adoption of cheaper and simple, yet robust, sampling protocols provide assurances for investors and project managers that it is possible to accurately assess carbon stocks in degraded and regenerating forests—thus helping to ensure that this opportunity is not missed.

## Supporting Information

S1 File Table and figures containing supporting analyses and information. Table A in S1 File. Correlation between the results of the relative contribution and the coefficient of variation of components of the forest carbon stocks in Paragominas and Santarém. Fig A in S1 File. Relationship between the carbon stocks stored in large (≥10cm DBH) and small (2–10cm DBH) live stems. Fig B in S1 File. Relationship between the carbon stocks stored in large live and dead stems (≥10cm DBH). Fig C in S1 File. Relationship between the carbon stocks stored in large live stems (≥10cm DBH) and small dead stems (2-10cm DBH). Fig D in S1 File. Relationship between the carbon stocks stored in large live stems (≥10cm DBH) and coarse woody debris. Fig E in S1 File. Relationship between the carbon stocks stored in large live stems (≥10cm DBH) and fine woody debris. Fig F in S1 File. Relationship between the carbon stocks stored in large live stems (≥10cm DBH) and leaf litter. Fig G in S1 File. Relationship between the carbon stocks stored in large live stems (≥10cm DBH) and in the first 30cm of soil. Fig H in S1 File. Evidence of understory fires found during field carbon assessments. Fig I in S1 File. Evidence of selective logging found during field carbon assessments.
(DOCX)
