# Supplementary material for: Developing Cost-Effective Field Assessments of Carbon Stocks in Human-Modified Tropical Forests
Source: PLoS One. 2015 Aug 26;10(8):e0133139. doi: 10.1371/journal.pone.0133139 (PMC4550286; doi:10.1371/journal.pone.0133139)
Supplement: S1 File — Table A in S1 File. Correlation between the results of the relative contribution and the coefficient of variation of components of the forest carbon stocks in Paragominas and Santarém. Fig A in S1 File. Relationship between the carbon stocks stored in large (≥10cm DBH) and small (2–10cm DBH) live stems. Fig B in S1 File. Relationship between the carbon stocks stored in large live and dead stems (≥10cm DBH). Fig C in S1 File. Relationship between the carbon stocks stored in large live stems (≥10cm DBH) and small dead stems (2-10cm DBH). Fig D in S1 File. Relationship between the carbon stocks stored in large live stems (≥10cm DBH) and coarse woody debris. Fig E in S1 File. Relationship between the carbon stocks stored in large live stems (≥10cm DBH) and fine woody debris. Fig F in S1 File. Relationship between the carbon stocks stored in large live stems (≥10cm DBH) and leaf litter. Fig G in S1 File. Relationship between the carbon stocks stored in large live stems (≥10cm DBH) and in the first 30cm of soil. Fig H in S1 File. Evidence of understory fires found during field carbon assessments. Fig I in S1 File. Evidence of selective logging found during field carbon assessments. (DOCX) [file pone.0133139.s001.docx]

**DEVELOPING COST-EFFECTIVE CARBON ASSESSMENTS IN HUMAN-MODIFIED TROPICAL FORESTS**

Erika Berenguer, Joice Ferreira, Toby A. Gardner, Luiz E. O. C. Aragão, Plínio Barbosa de Camargo, Carlos Eduardo Cerri, Mariana Durigan, Raimundo Cosme de Oliveira Junior, Ima Célia Guimarães Vieira, Jos Barlow

**Electronic supplementary information**

Table A. **Correlation between the results of the relative contribution and the coefficient of variation of components of the forest carbon stocks in Paragominas and Santarém.** Results are separated into three hypothetical scenarios of carbon stocks assessments in human-modified tropical forests: 1) No *a priori* information of forest class; 2) Primary forests only – includes undisturbed and disturbed primary forests; and 3) Secondary forests only.

| **Scenario** | **Relative contribution** | | **Coefficient of variation** | |
| --- | --- | --- | --- | --- |
|  | **rho** | ***p*** | **rho** | ***p*** |
| No *a priori* information of forest class | 0.976 | <0.001 | 0.905 | 0.004 |
| Primary forests only | 1.000 | <0.001 | 0.905 | 0.005 |
| Secondary forests only | 0.952 | 0.001 | 0.810 | 0.022 |


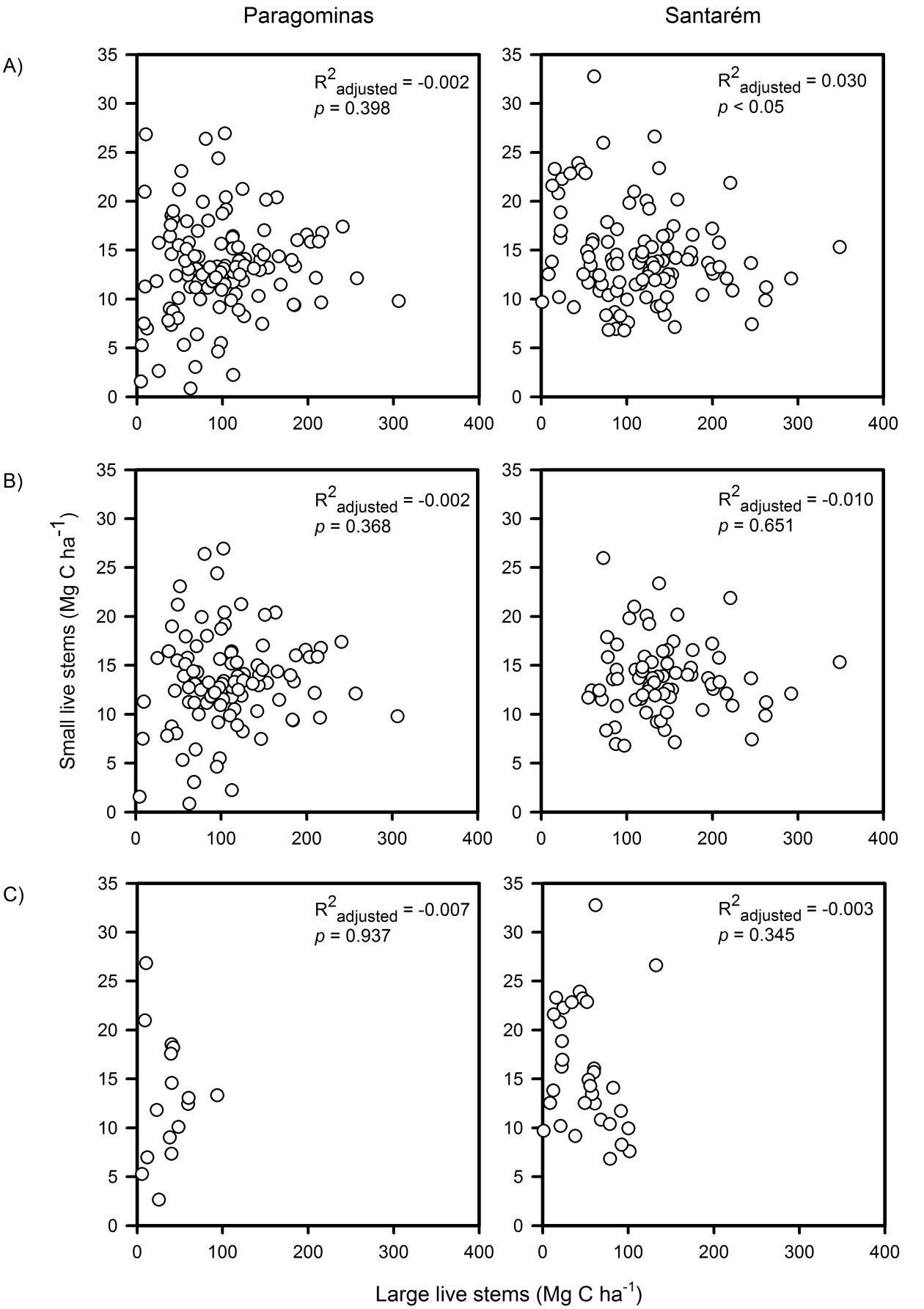


Fig.A. **Relationship between the carbon stocks stored in large (≥10cm DBH) and small (2-10cm DBH) live stems.** Results are separated into three hypothetical scenarios of carbon stock assessments in human-modified tropical forests: A) No *a priori* information of forest class; B) Primary forests only – includes undisturbed and disturbed primary forests; and C) Secondary forests only.


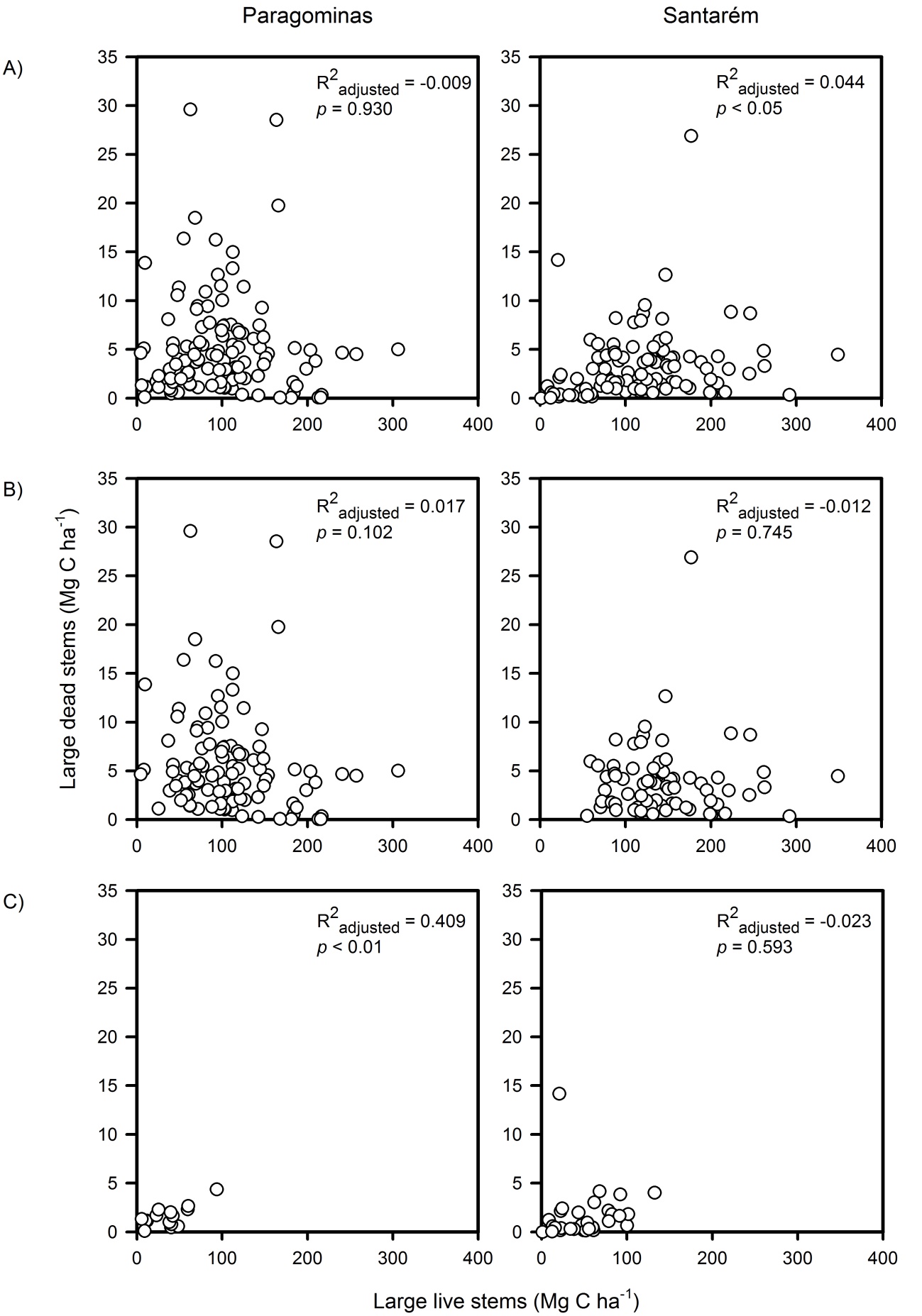


Fig.B. **Relationship between the carbon stocks stored in large live and dead stems (≥10cm DBH).** Results are separated into three hypothetical scenarios of carbon stock assessments in human-modified tropical forests: A) No *a priori* information of forest class; B) Primary forests only – includes undisturbed and disturbed primary forests; and C) Secondary forests only.


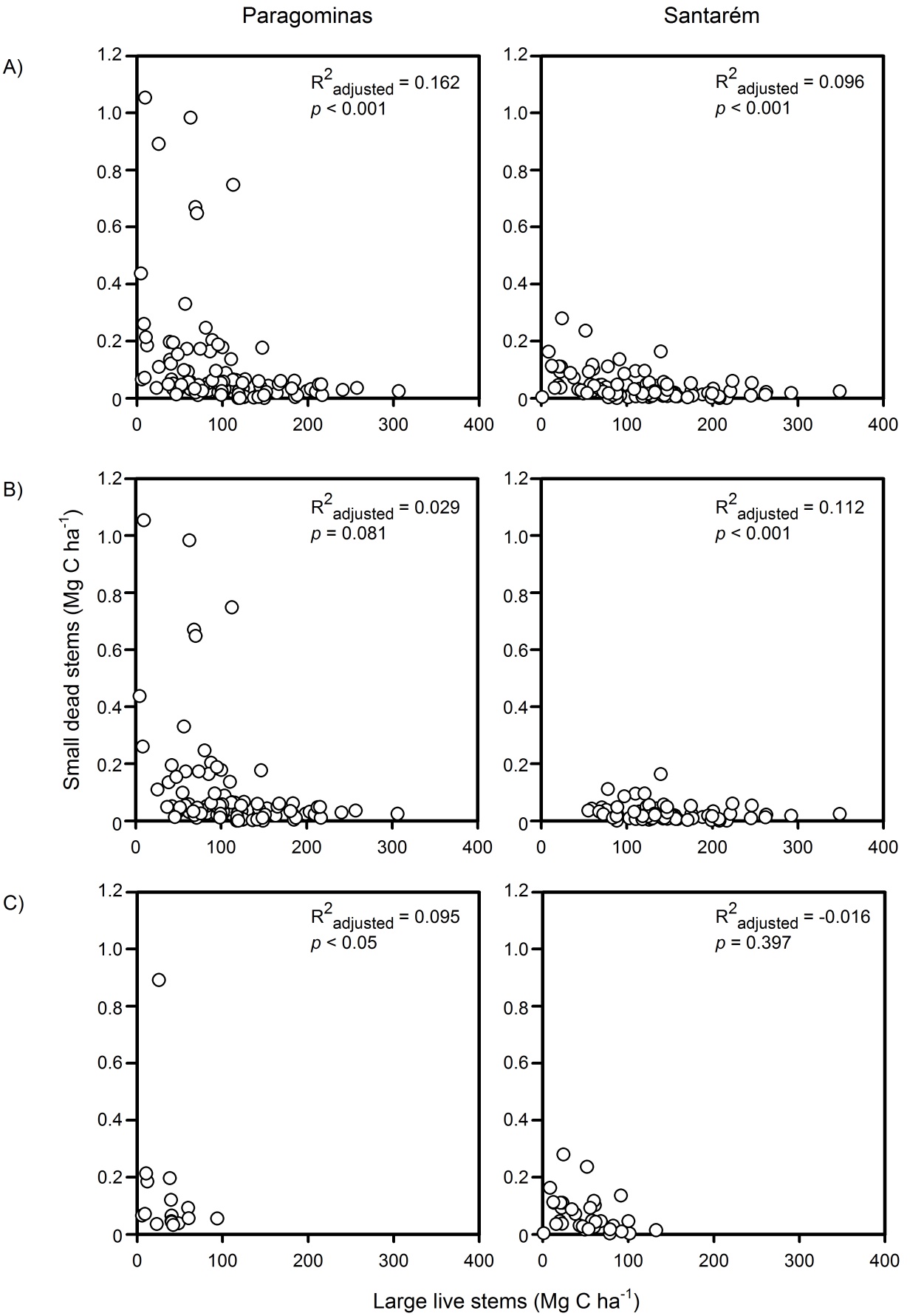


Fig.C. **Relationship between the carbon stocks stored in large live stems (≥10cm DBH) and small dead stems (2-10cm DBH).** Results are separated into three hypothetical scenarios of carbon stock assessments in human-modified tropical forests: A) No *a priori* information of forest class; B) Primary forests only – includes undisturbed and disturbed primary forests; and C) Secondary forests only.


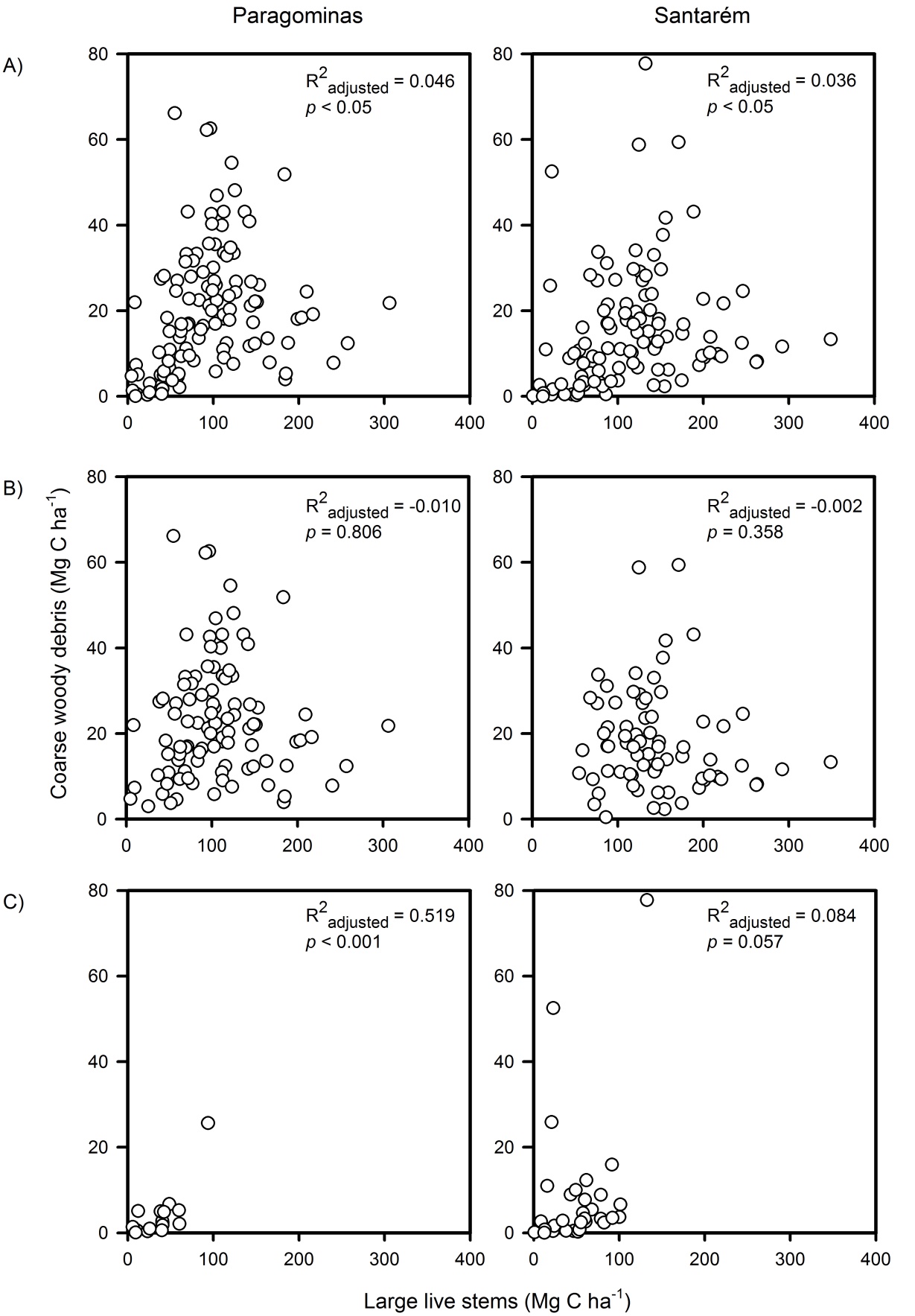


Fig.D. **Relationship between the carbon stocks stored in large live stems (≥10cm DBH) and coarse woody debris.** Results are separated into three hypothetical scenarios of carbon stock assessments in human-modified tropical forests: A) No *a priori* information of forest class; B) Primary forests only – includes undisturbed and disturbed primary forests; and C) Secondary forests only.


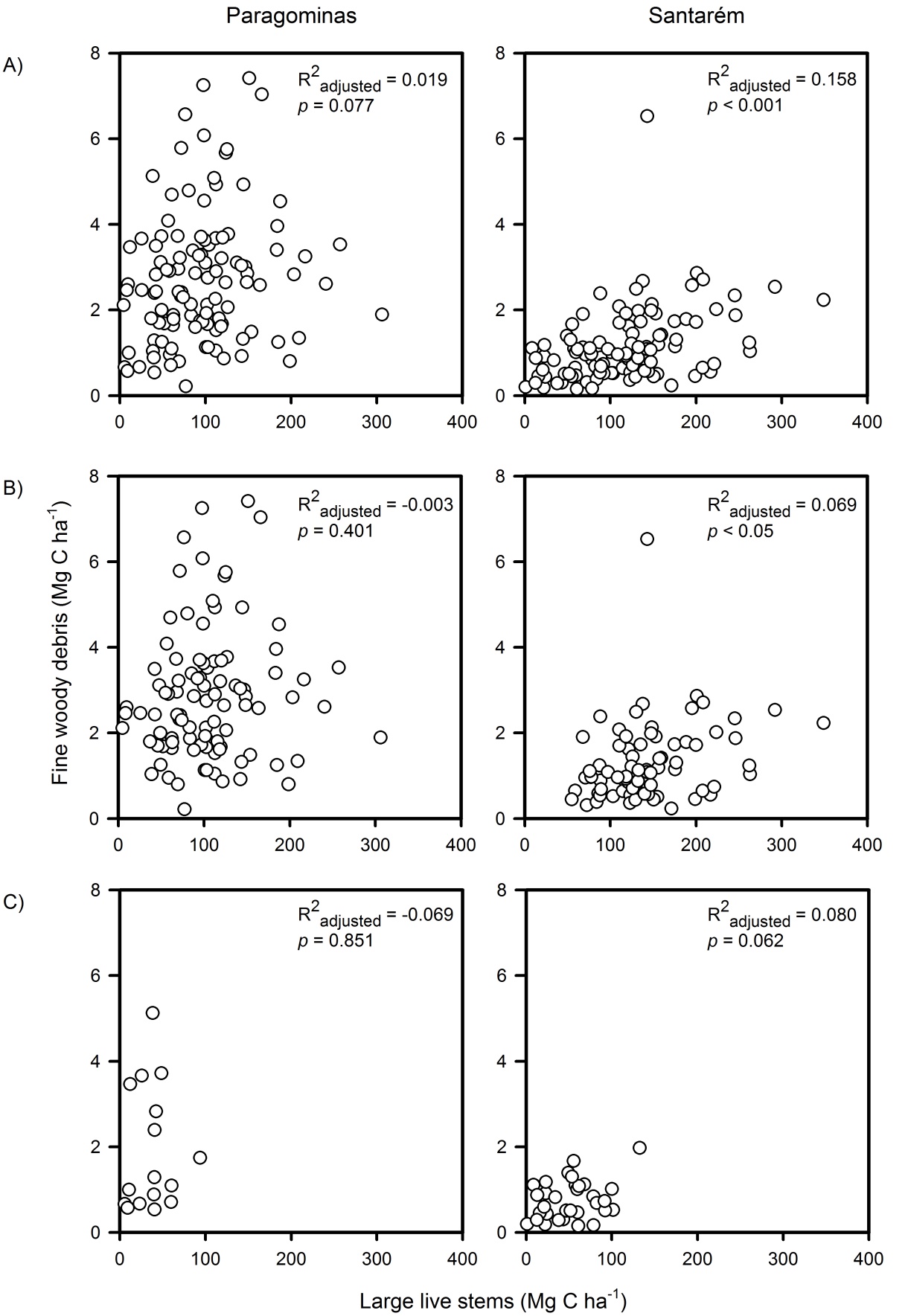


Fig.E. **Relationship between the carbon stocks stored in large live stems (≥10cm DBH) and fine woody debris.** Results are separated into three hypothetical scenarios of carbon stock assessments in human-modified tropical forests: A) No *a priori* information of forest class; B) Primary forests only – includes undisturbed and disturbed primary forests; and C) Secondary forests only.


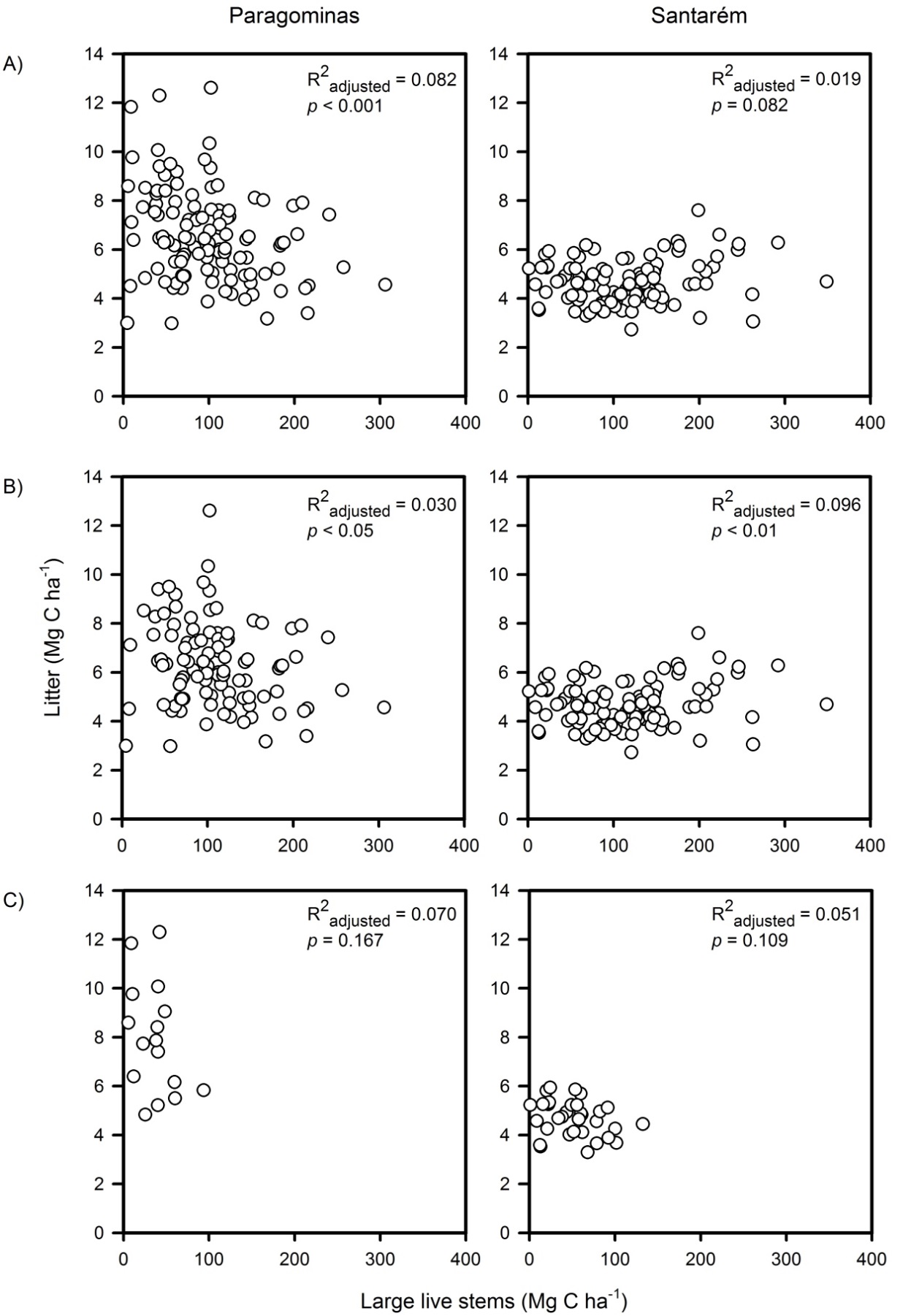


Fig.F. **Relationship between the carbon stocks stored in large live stems (≥10cm DBH) and leaf litter.** Results are separated into three hypothetical scenarios of carbon stock assessments in human-modified tropical forests: A) No *a priori* information of forest class; B) Primary forests only – includes undisturbed and disturbed primary forests; and C) Secondary forests only.


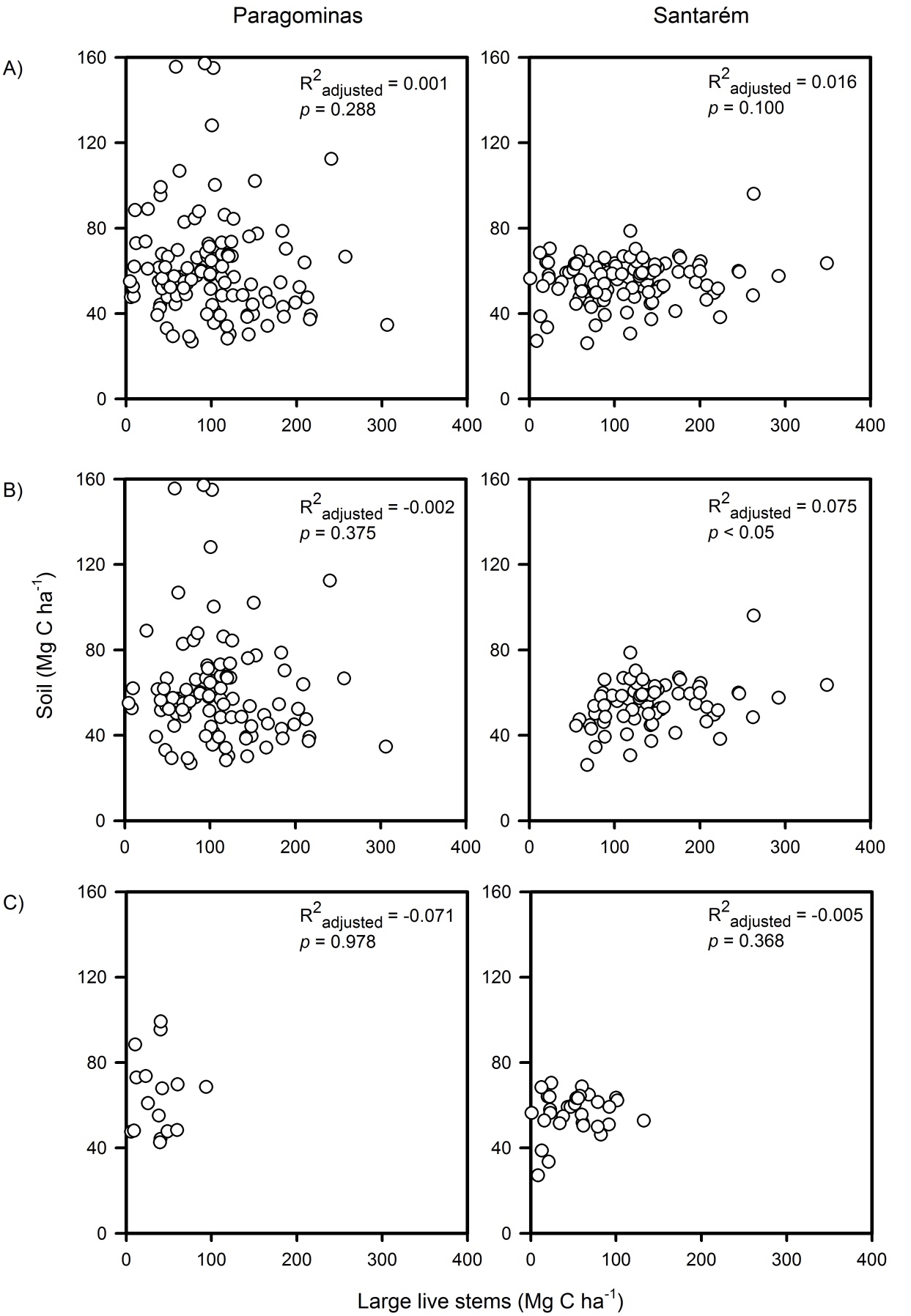


Fig.G. **Relationship between the carbon stocks stored in large live stems (≥10cm DBH) and in the first 30cm of soil.** Results are separated into three hypothetical scenarios of carbon stock assessments in human-modified tropical forests: A) No *a priori* information of forest class; B) Primary forests only – includes undisturbed and disturbed primary forests; and C) Secondary forests only.


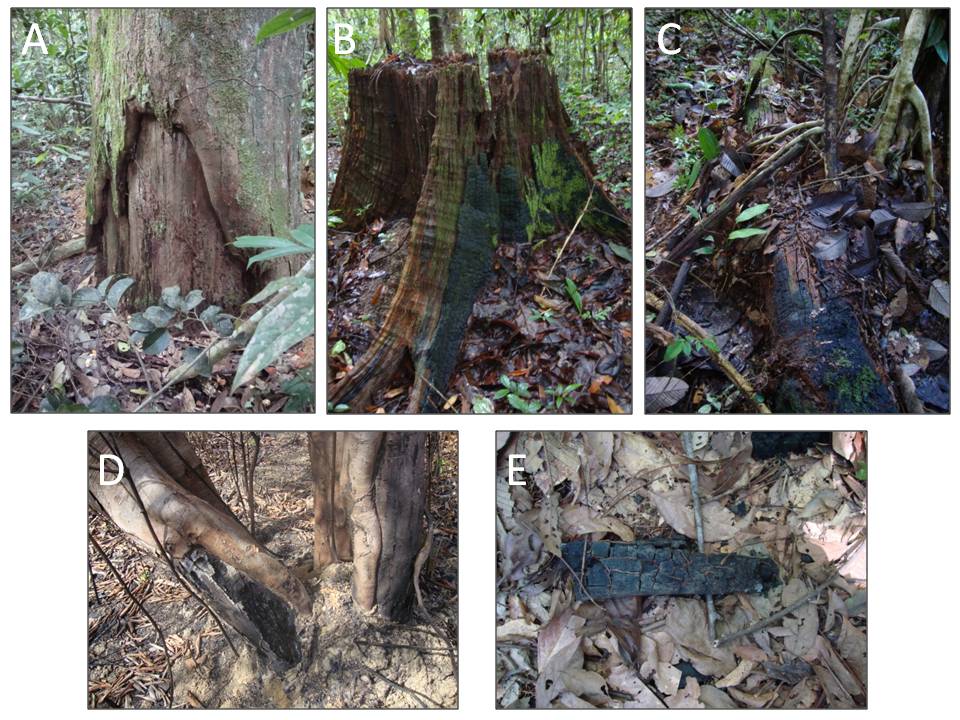


Fig.H. **Evidence of understory fires found during field carbon assessments.** A) Fire scar on a live stem, B) Charred logged stump, C) Charred coarse woody debris, D) Charred fire scar on live stems and presence of ash on the forest floor, E) Charcoal found on the forest floor. All photos taken in Paragominas and Santarém by E.B.


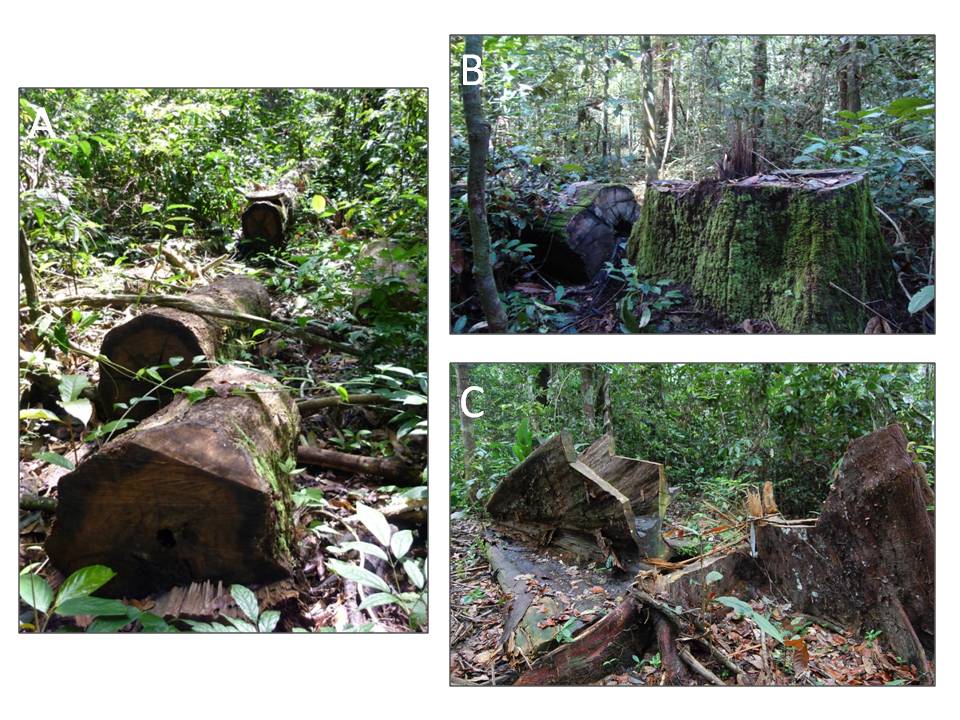


Fig.I. **Evidence of selective logging found during field carbon assessments.** A) Leftover logs, B) Logged tree stump and leftover log, C) Leftover buttresses from a logged tree. All photos taken in Paragominas and Santarém by E.B.
